# Follicle Stimulating Hormone and Anti-Müllerian Hormone among Fertile and Infertile Women in Ile-Ife, Nigeria: Is there A Difference?

**DOI:** 10.22074/ijfs.2016.4645

**Published:** 2016-11-11

**Authors:** Temitope Okunola, Kayode Olusegun Ajenifuja, Olabisi Morebise Loto, Afolabi Salawu, Stephen Oluseyi Omitinde

**Affiliations:** 1 Department of Obstetrics and Gynecology, Obafemi Awolowo University Teaching Hospitals Complex, Ile-Ife, Nigeria; 2 Obafemi Awolowo University, Ile-Ife, Nigeria; 3 Ladoke Akintola University of Technology Teaching Hospital, Ogbomoso, Nigeria

**Keywords:** Infertility, Ovarian Reserve, Follicle Stimulating Hormone, Anti-Müllerian Hormone

## Abstract

**Background:**

Reduced ovarian reserve predicts poor ovarian response and poor suc- cess rates in infertile women who undergo assisted reproductive technology (ART). Ovarian reserve also decreases with age but the rate of decline varies from one woman to another. This study aims to detect differences in ovarian reserve as measured by basal serum follicle stimulating hormone (FSH) and anti-Müllerian hormone (AMH) between a matched cohort of fertile and infertile regularly menstruating women, 18-45 years of age.

**Materials and Methods:**

This case-control study involved 64 fertile and 64 subfertile women matched by age at recruitment. Peripheral blood samples were taken from the women recruited from the Gynecological and Outpatient Clinics of Obafemi Awolowo University Teaching Hospital, Ile-Ife, Nigeria. Serum FSH and AMH were quantified using ELISA at the Metabolic Research Laboratory of LAUTECH Teaching Hospital, Ogbomoso, Nigeria.

**Results:**

A significant difference existed in the mean FSH of fertile (6.97 ± 3.34) and infertile (13.34 ± 5.24, P=0.013) women. We observed a significant difference in AMH between fertile (2.71 ± 1.91) and infertile (1.60 ± 2.51, P=0.029) women. There was a negative correlation between FSH and AMH in both fertile (r=-0.311, P=0.01) and infertile (r=-0.374, P=0.002) women.

**Conclusion:**

The difference in ovarian reserve observed in this study suggests that reduced ovarian reserve in regularly menstruating women may be associated with early ovarian ageing or subfertility.

## Introduction

Infertility is the inability of a couple to conceive despite adequate unprotected sexual intercourse within one year (1). Infertility affects 10-30% of couples in sub-Saharan Africa (2, 3). Infertility and its management place substantial psychosocial demand on the couple, especially the woman (4). The physician therefore needs to be well equipped in order to manage couples with infertility.

The number of oocytes in a female reaches its peak at 20 weeks during fetal life at 7 million primordial follicles. At birth, human ovaries contain approximately 1 million primordial follicles which arrest at the prophase of the first meiotic division (5, 6). This further reduces to 400,000 at puberty and only about 400 follicles will eventually acquire gonadotrophin receptors and the possibility of ovulation. Follicle depletion occurs before and after menarche, during use of oral contraceptives, pregnancy, and whether menstruation is regular or not. As the depletion of the follicular pool continues during the reproductive life, there is regular escape of the primordial follicles from the resting phase by entering into meiosis (6).

Longitudinal studies in fertile women have shown declines in anti-Müllerian hormone (AMH) levels with age; it is the earliest marker of decline in ovarian reserve in young women (7). The purposes of assessing ovarian reserve are to predict reproductive age; detect early ovarian ageing (currently affecting 10% of the general population); predict chances for conception in women desirous of pregnancy; and in counseling women desirous of delaying childbearing (8, 9). There is a large individual variability that exists in age at which ovarian aging commences. Factors that contribute to biological ovarian aging and reduction in ovarian reserve include ovarian toxicants, chromosomal abnormality, cigarette smoking, alcohol abuse, nutritional deficiencies, oxidative stress, and auto-immunity. Gynecological conditions and treatments such as pelvic surgery, chemotherapy, and radiotherapy also affect the rate of decline in ovarian reserve (10). The possibility thereby exists that exposure to the factors that accelerate ovarian aging associated with reduction in ovarian reserve is associated with subfertility. 

The aim of this study was to detect differences in ovarian reserve as assessed by AMH and follicle stimulating hormone (FSH) between fertile and infertile women in Ile-Ife, Nigeria. We hypothesized that a difference exists in ovarian reserve as measured by basal serum FSH and random serum AMH levels in infertile women compared to fertile women.

## Materials and Methods

### Study population and participants

A study by Kalaiselvi et al. (11) compared mean AMH levels between fertile women (3.7 ± 1.6 ng/ ml) and subfertile women (2.9 ± 0.98 ng/ml); this was used to calculate the sample size according to the formula for comparison of means (12). Assuming a minimum detectable difference of 0.8 ng/ml, 95% confidence interval (CI), study power of 90% with attrition rate of 10%, we required 65 participants in each group to ensure statistically significant results. This case-control study enrolled 65 subfertile women recruited from the Gynecology Clinic of Obafemi Awolowo University Teaching Hospitals Complex, Ile-Ife, Nigeria and 65 fertile women matched by age with the infertile group recruited from the general Outpatient Clinic of this hospital from November 2014 to January 2015. All women recruited were between the ages of 18 and 45 years and had regular menstrual cycles that ranged from 21 to 35 days. The fertile participants also had proven natural fertility with at least one pregnancy carried to term within the preceding 2 years; each pregnancy haven arisen spontaneously following unprotected sexual intercourse within 1 year. Subfertile participants had at least a 12-month history of inability to conceive despite adequate sexual intercourse. We excluded women with any history, radiological or biochemical parameters suggestive of polycystic ovary syndrome (PCOS) or evidence of endocrinological diseases, and those that used hormonal contraceptives. 

The study proforma was then completed to document demographic and gynecological information. Study outcomes included basal serum FSH and random serum AMH levels. A venous blood sample was taken for serum AMH measurement. Each woman was instructed to alert the investigator at the onset of her next menstrual cycle in order to make arrangement for collection of the day 3 FSH sample. Peripheral blood samples were collected through a venipuncture by a doctor who collected 5 ml for each assay. Samples were collected into plain sterile sample bottles and left to stand for 1 hour for clot retraction and then centrifuged for 10 minutes at 5000 rpm. Serum was then separated into another unheparinized sterile sample using a pipette. The serum was then stored in a -20ºC freezer until analysis within 3 weeks. The samples were transported to the laboratory in ice packs.

### Follicle stimulating hormone and anti-Müllerian hormone assays

Serum samples were thawed at room temperature and processed at the Metabolic Research Laboratory of Ladoke Akintola University of Technology (LAUTECH) Teaching Hospital, Ogbomoso. Serum FSH was quantified in duplicate with Follicle Stimulating Hormone Test System (Monobind, Inc., USA) using the direct enzyme linked immunosorbent (ELISA) assay according to the manufacturer’s manual. After incubation, the absorbance was read at 450 nm within 30 minutes using a microplate ELISA reader (LT 4000). The precision of the assay was 0.134 mIU/ml.

AMH was quantified in duplicate with the Human Anti-Müllerian Hormone ELISA kit (Span Biotech Ltd., Hong Kong) using a double-antibody sandwich ELISA according to the manufacturer’s manual. After incubation, the absorbance was read as above. The sensitivity of the assay was 0.01 ng/ml.

### Statistical analysis

We analyzed data from 128 women with Stata version 13 (StataCorp). Pearson’s correlation was used to determine the relationship between age, body mass index (BMI), AMH, and FSH while the paired t-test was used to compare means between the two groups.

### Ethical consideration

The Ethics and Research Committee of Obafemi Awolowo University Teaching Hospitals Complex, Ile-Ife approved the study (Ethical clearance certificate number: ERC/2014/05/01). Informed consent was obtained from each participant before enrollment.

## Results

We recruited 130 women into the study from November 2014 to January 2015; sixty five women in the subfertile group and sixty five women in the fertile group. However, two participants, one from each group, did not complete the study. Therefore, we analyzed the data from 128 women who completed the study.

### Baseline characteristics

We compared the baseline characteristics of the recruited women between the two groups (Table 1). The fertile group had a mean age of 31.16 ± 5.78 years; for the subfertile group, the mean age was 31.52 ± 4.35 years. The mean age difference between the two groups was 0.36 (P=0.58, 95% CI: 4.65-0.88). The mean BMI of the fertile group was 26.31 ± 4.48 vs. 26.03 ± 5.74 for the subfertile group. The mean difference in BMI difference between the two groups was 0.27 (P=0.77, 95% CI: 1.45-1.99). The mean parity of the fertile group was 1.95 ± 1.08 compared to 0.48 ± 0.97 for the subfertile group. The mean difference in parity was 1.48 (P=0.00, 95% CI: 1.12-1.83, Table 1).

Among the subfertile women, 27 (42.2%) had primary infertility while 37 (57.8%) had secondary infertility. There were 44 (68.8%) women in the subfertile group diagnosed with tubal factor infertility. There were 5 (7.8%) anovulatory cases and 4 (6.3%) with male factor infertility. Both tubal and male factors were present in one (1.6%) participant while another participant (1.6%) also had both tubal factor and anovulation. A third participant (1.6%) had both male factor and anovulation. However, 15 (23.4%) remained unexplained at the conclusion of the study. Participants aged 18-24 years, 25-34 years and 35-45 years were 6, 39 and 20 respectively.

**Table 1:** Comparison of age, parity, and body mass index (BMI) between fertile and subfertile women


	Fertile (mean ± SD)	Infertile (mean ± SD)	Mean difference	95% CI	t statistics	P value

Age (Y)	31.16 ± 5.78	31.52 ± 4.35	-0.36	-4.65-0.88	0.83	0.58
BMI (kg/m^2^)	26.31 ± 4.48	26.03 ± 5.74	0.27	-1.45-1.99	0.31	0.77
Parity	1.95 ± 1.08	0.48 ± 0.97	1.48	1.12-1.83	8.20	0.00*


CI; Confidence interval and *; Statistically significant.

### Correlation of ovarian reserve markers in fertile women

**Fig.1:**
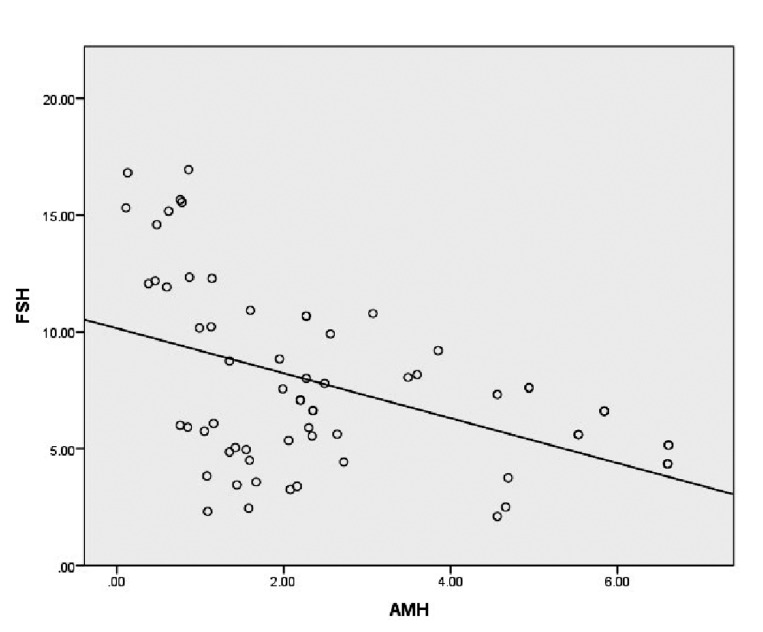
Relationship between FSH and AMH among fertile women. FSH; Follicle stimulating hormone and AMH; Anti-Müllerian hormone.

Fertile women had moderately negative correlations between FSH and AMH, as well as AMH and age whereas we observed a positive correlation between age and FSH (Table 2). However, neither FSH nor AMH had any significant association with BMI (Table 2). Figure 1 depicts the association between FSH and AMH. The Pearson’s rho coefficient for the correlation between FSH and AMH after controlling for age was -0.24 (P=0.04).

**Table 2:** Correlation between anti-Müllerian hormone (AMH), follicle stimulating hormone (FSH), body mass index (BMI), and age in fertile women


Parameters	FSH		AMH	
	Pearson correlation coefficient	P value	Pearson correlation coefficient	P value

Age (Y)	0.258	0.038*	-0.332	0.007*
BMI (kg/m^2^)	0.14	0.28	-0.044	0.726
FSH (IU/L)	1	-	-0.311	0.01*


*; Statistically significant.

### Correlation of ovarian reserve markers in subfertile women

**Fig.2:**
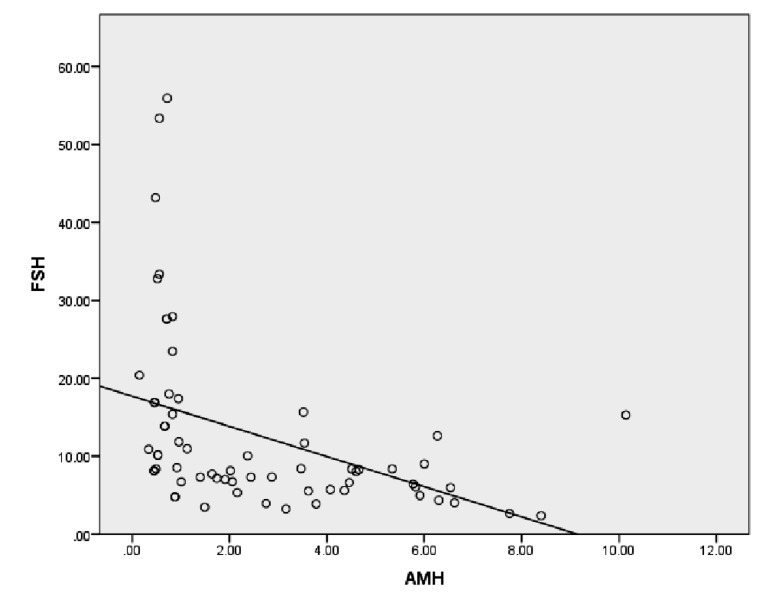
Relationship between FSH and AMH among infertile women. FSH; Follicle stimulating hormone and AMH; Anti-Müllerian hormone.

Subfertile women had negative correlations between FSH and AMH, as well as between AMH and age. A positive correlation existed between age and FSH (Table 3). Also, neither FSH nor AMH had any significant association with BMI (Table 3). Figure 2 depicts the association between FSH and AMH. The Pearson’s rho coefficient for the correlation between FSH and AMH after controlling for age was -0.311 (P=0.012).

**Table 3:** Correlation between anti-Müllerian hormone (AMH), follicle stimulating hormone (FSH), body mass index (BMI), and age in subfertile women


Parameters	FSH		AMH	
	Pearson correlation coefficient	P value	Pearson correlation coefficient	P value

Age (Y)	0.292	0.01*	-0.323	0.009*
BMI (kg/m^2^)	0.01	0.93	0.005	0.972
FSH (IU/L)	1	-	-0.374	0.002*


*; Statistically significant.

**Table 4:** Comparison of anti-Müllerian hormone (AMH) and follicle stimulating hormone (FSH) between fertile and infertile women


	Fertile (mean ± SD)	Infertile (mean ± SD)	Mean difference	95% CI	t statistics	P value

FSH (IU/L)	6.97 ± 3.34	13.34 ± 5.24	-6.37	-11.36- -1.38	-2.55	0.013*
AMH (ng/ml)	2.71 ± 1.91	1.60 ± 2.51	1.11	1.06-1.83	1.21	0.029*


CI; Confidence interval and *; Statistically significant.

**Table 5:** Sub-analysis by age groups


Age (Y)		Fertile (mean ± SD)	Infertile (mean ± SD)	Mean difference	95% CI	t statistics	P value

25-34	FSH (IU/L)	6.26 ± 2.25	7.41 ± 7.85	-0.97	-5.94-3.38	-0.45	0.65
	AMH (ng/ml)	3.20 ± 1.84	1.37 ± 2.63	1.82	0.36-2.72	1.52	0.043*
35-45	FSH (IU/L)	8.87 ± 4.46	28.48 ± 10.42	19.60	6.67-32.20	-3.20	0.004*
	AMH (ng/ml)	1.76 ± 1.92	0.83 ± 2.31	1.08	0.43-3.66	1.49	0.031*


CI; Confidence interval, FSH; Follicle stimulating hormone, AMH; Anti-Müllerian hormone, and *; Statistically significant.

**Table 6:** Sub-analysis using clinical groups


		Fertile (mean ± SD)	Infertile (mean ± SD)	Mean difference	95% CI	t statistics	P value

Tubal factor	FSH (IU/L)	7.16 ± 3.53	9.48 ± 12.80	-2.32	-4.97--2.32	-2.73	0.19*
	AMH (ng/ml)	2.64 ± 1.84	1.55 ± 2.77	1.10	0.54-1.99	1.28	0.024*
Unexplained	FSH (IU/L)	6.67 ± 2.59	19.53 ± 15.91	-12.74	-27.36-1.87	-2.87	0.043*
	AMH (ng/ml)	2.97 ± 2.29	2.14 ± 2.15	0.83	-0.88-2.53	1.03	0.32


CI; Confidence interval, FSH; Follicle stimulating hormone, AMH; Anti-Müllerian hormone, and *; Statistically significant.

### Comparison of ovarian reserve markers between fertile and subfertile women

Fertile women had a mean FSH value of 6.97 ± 3.34, whereas this value was 13.34 ± 5.24 for subfertile women. The mean difference was -6.37 (P=0.013, 95% CI:-11.36 to-1.38). The fertile group had a mean AMH value of 2.71 ± 1.91. The subfertile group had a mean AMH value of 1.60 ± 2.51. Their mean difference was 1.11 (P=0.029, 95% CI: 1.06 to 1.83;,Table 4).

Sub analysis performed after categorizing the participants into age groups showed significant differences in both mean FSH and AMH levels in women aged 35-45 years, while only AMH showed a significant difference in women aged 25-35 years (Table 5). 

Women segregated according to clinical conditions showed that tubal factor forms the majority of cases A statistically significant difference existed between the mean FSH and AMH levels in women with tubal factor infertility, whereas serum AMH did not differ in patients with unexplained infertility (Table 6).

## Discussion

This research work showed significantly higher basal serum FSH and lower random serum AMH levels in subfertile women compared to fertile women in Ile Ife, Southwestern Nigeria. The strength of this study was the participation of both young and older women. However, the hormonal levels did not correlate with number of oocytes retrieved, pregnancy rate, or live births. In addition, we did not include other ovarian reserve markers such as antral follicle count in the study.

No statistically significant difference existed in the mean age and BMI between the fertile and subfertile groups. The subfertile group had significantly lower parity. We have expected this finding because it is the major difference between these two groups. Zaidi et al. (9) reported a significant difference in the BMI among the older fertile and subfertile women aged 30-39 years. The discrepancy between this study and other studies might be due to the difference in the age groups compared in both studies. The result obtained here, however, was comparable to the study by Kalaiselvi et al. (11).

There was a moderate negative correlation between FSH and AMH among the fertile women, which was similar to the reports (13). Random serum AMH level reduced as the basal serum FSH increased. This could be explained by the fact that increased basal serum FSH and reduced random serum AMH depicted a decline in ovarian reserve which tended to occur with increasing age. However, a stronger positive correlation between age and FSH was reported by another study; this might be attributed to a larger sample size (14). BMI did not correlate significantly with both basal FSH and random AMH which was comparable to findings from other studies (15, 16).

The negative correlation between AMH and age among the subfertile group compared to other studies in infertile women (17, 18). In this study, the basal serum FSH increased with increased age. There was no correlation between AMH, FSH, and BMI among the subfertile women. There were conflicting reports about the correlation between BMI and ovarian reserve tests in subfertile women such as the study by Buyuk et al. (19) that reported lower serum AMH levels among overweight and obese women with reduced ovarian reserve.

Subfertile women had statistically significant higher basal serum FSH levels which compared to the results reported by Kalaiselvi et al. (11). This further corroborated the findings by other researchers that reported a decline in ovarian reserve among regularly menstruating infertile women (11, 20). Erdem et al. (21) however did not find any difference in basal serum FSH between fertile and subfertile women. This might be due to patient selection in their study, which consisted of older women.

In addition, random serum AMH also differed significantly between the two groups of women. We observed significantly lower random serum AMH in the subfertile women. This supported other studies about AMH (11, 22). Kalaiselvi et al. (11) reported significantly lower AMH in subfertile women. This difference in AMH between both groups also supported a decline in ovarian reserve in subfertile women. Therefore, ovarian reserve might be reduced in regularly menstruating subfertile women.

Younger infertile women had reduced AMH and normal serum FSH levels, whereas older infertile women had both reduced AMH and elevated FSH levels. This suggested that older women with reduced ovarian reserve were more likely to show both elevated FSH and reduced AMH levels while younger women with diminished ovarian reserve were likely to have normal FSH but reduced AMH levels. This finding supported previous studies where elevated FSH was a late indicator of diminished ovarian reserve (23).

Mean serum AMH did not differ among the unexplained infertility group, whereas we have observed a difference in mean serum FSH levels. This could be due to the fact that serum FSH is secreted from the anterior pituitary and depends on other factors such as serum estrogen while AMH is secreted directly from the preantral follicles (24). Women with unexplained infertility may therefore have other factors responsible for elevated FSH levels.

## Conclusion

Ovarian reserve, as assessed by basal serum FSH and random serum AMH, significantly reduced in regularly menstruating subfertile women. A statistically significant difference existed in ovarian reserve of infertile women compared to fertile women in Ile-Ife, Nigeria. Therefore, reduction in ovarian reserve might be associated with early ovarian ageing or subfertility. 
